# Impression cytology of ocular surface in xeroderma pigmentosum

**DOI:** 10.5935/0004-2749.2023-0026

**Published:** 2024-03-27

**Authors:** Allexya Affonso Antunes Marcos, Denise Freitas, Jeison de Nadai Barros, Arthur Gustavo Fernandes, Márcia Lowen, Moacyr Rigueiro, Melina Correia Morales, Rubens Belfort Neto, Arun D. Singh

**Affiliations:** 1 Department of Ophthalmology and Visual Sciences, Hospital São Paulo, Escola Paulista de Medicina, Universidade Federal de São Paulo, São Paulo, SP, Brazil; 2 Department of Pathology, Escola Paulista de Medicine, Universidade Federal de São Paulo, São Paulo, SP, Brazil; 3 Department of Ophthalmology, Cleveland Clinic, Cleveland, US

**Keywords:** Xeroderma pigmentosum, Eye neoplasms, Conjunctiva/cytology, Cornea/cytology, Cytological techniques

## Abstract

**Purpose:**

To describe cellular alterations detected by impression cytology of the ocular surface in patients with xeroderma pigmentosum. The secondary objective was to assess the reliability of impression cytology in diagnosing ocular surface squamous neoplasia.

**Methods:**

Patients with xeroderma pigmentosum underwent a single-day complete ophthalmological examination and impression cytology for ocular surface evaluation using 13 mm diameter mixed cellulose esters membrane filters and combined staining with Periodic Acid Schiff, Hematoxylin and Eosin, and Papanicolaou stains followed by microscopic analysis. The cytological findings were correlated with the clinical diagnosis. The impression cytology findings at baseline and one-year follow-up were correlated with the clinical course (no tumor, treated tumor, residual tumor recurrent tumor, new tumor).

**Results:**

Of the 42 patients examined, impression cytology was performed in 62 eyes of 34 participants (65% females). The mean age of patients was 29.6 ± 17 years (range 7-62). Fifteen eyes had a clinical diagnosis of ocular surface squamous neoplasia. Impression cytology showed goblet cells (47, 75%), inflammatory cells (12, 19%), keratinization (5, 8%), and squamous metaplasia (30, 48%). Impression cytology was positive for atypical cells in 18 patients (12 with and 6 without ocular surface squamous neoplasia). The sensitivity, specificity, positive predictive value, and negative predictive value of impression cytology (at baseline) for diagnosis of ocular surface squamous neoplasia were 80%, 87%, 67%, and 93%, respectively, using clinical diagnosis of ocular surface squamous neoplasia as the reference standard.

**Conclusion:**

Impression cytology has a moderate positive predictive value for the diagnosis of ocular surface squamous neoplasia in patients with xeroderma pigmentosum. However, the lack of detection of atypical cells on impression cytology has a high negative predictive value for ocular surface squamous neoplasia. Integration of impression cytology in the long-term management of high-risk patients, such as patients with xeroderma pigmentosum, can avoid unnecessary diagnostic biopsies.

## INTRODUCTION

Xeroderma pigmentosum (XP) is a rare type of genodermatosis inherited as an autosomal recessive condition. It is characterized by hypersensitivity to sunlight resulting in exaggerated photoaging and photocarcinogenesis^(1^-^3)^. Compared to the general population, XP patients aged up to 20 years have a 1000-fold higher risk of cancer of the sun-exposed tissues of the eye^(2)^.

Impression cytology (IC) is a technique for sampling the superficial layers of the ocular surface by applying collecting devices. Cells adhering to the surface of the device can be removed and processed further for analysis by a variety of methods. It is a simple and noninvasive technique for diagnosis and posttreatment follow-up of several ocular surface disorders^(4^,^5)^. IC is helpful for the evaluation of several conditions such as dry eye syndrome, cicatrizing conjunctivitis, vitamin A deficiency, limbal stem cell failure, effects of various medications, and ocular surface malignant tumors^(6^-^13)^.

This study aimed to perform a descriptive analysis of the cellular alterations observed by IC of the ocular surface in patients with XP. The secondary objective was to assess the reliability of IC in diagnosing ocular surface squamous neoplasia (OSSN).

## METHODS

This was a longitudinal study conducted from October 2018 to October 2019 to evaluate cellular changes in XP patients by performing IC for assessment of the ocular surface. Patients were recruited via social media (Facebook^®^, Instagram^®^) and messaging apps such as WhatsApp^®^. The study was approved by the Institutional Review Board of the CEP Universidade Federal de São Paulo - Unifesp, (approval number CAAE: 95105818.7.0000.5505). Written informed consent of patients was obtained prior to their enrolment.

### Clinical assessment

The diagnosis of OSSN was based on slit-lamp examination and ancillary studies such as the assessment of the staining pattern of 1% toluidine blue (TB) eye drops. The TB staining patterns were categorized into 2 groups: “homogeneous” (score 1) and “stippled” (dot-shaped) (score A homogenous staining pattern was defined as the presence of homogeneous TB staining without stippled appearance). The distribution of dye uptake was then classified as follows: scattered (score 1); focal patches (score 2); and diffuse (score 3). Total scores ≤4 were considered “negative TB staining” and scores ≥5 were considered “positive TB staining.” In case of any inter-observer disagreement regarding staining interpretation, the consensus opinion was selected as the final staining result. Additionally, TB-stained areas that also stained positive with fluorescein dye were not considered positive for TB staining^(14)^ and anterior segment optical coherence tomography (OCT) pattern (severely thickened and hyperreflective epithelium with an abrupt transition between the normal and affected epithelium) was considered positive for OSSN^(15)^.

### IC: procedure

After administration of topical anesthesia with 0.5% deacaine hydrochloride (Anestalcon 0.5%, Alcon, São Paulo, Brazil), a strip of cellulose acetate filter paper with a diameter of 13 mm and pore size of 0.45 mm (Millipore HAWP01300, Bedford, US) was placed on the patient’s ocular surface, pressed gently for 5 seconds, and then removed. The filter was immediately fixed for approximately 10 min in a solution containing glacial acetic acid, 37% formaldehyde, and ethyl alcohol in a volume ratio of 1:1:20. IC was performed on both eyes, when possible. In patients with a visible OSSN lesion, the filter paper was applied over the lesion. Otherwise, samples were collected from the nasal and temporal bulbar conjunctiva. Patients who underwent one-year follow-up examination were sampled at the same site as the baseline IC.

### IC: interpretation

**Figure 1:**
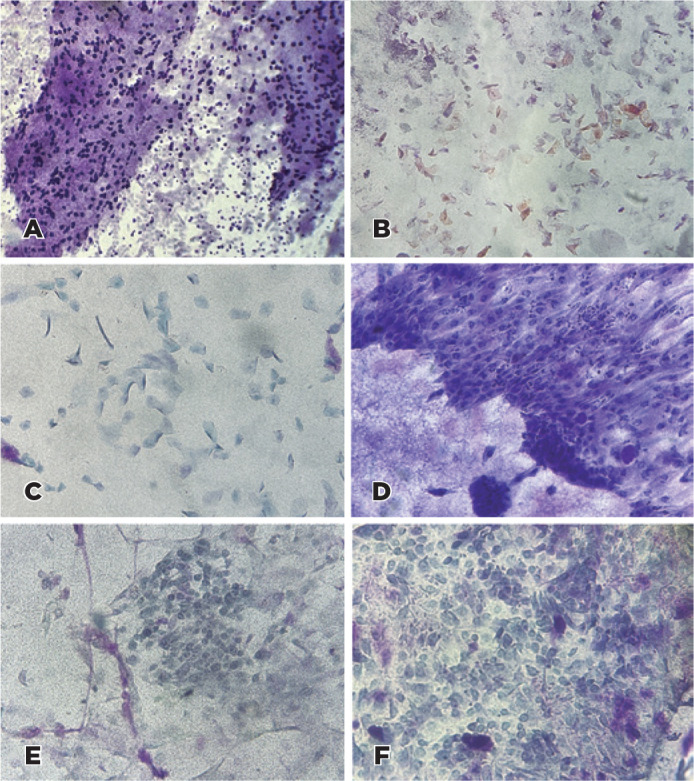
Impression cytology for the evaluation of the ocular surface of patients with Xeroderma Pigmentosum. Goblet cells (PAS, Hematoxylin-eosin staining, original magnification 100×) (A); Keratinization (PAS, Hematoxylin-eosin staining, original magnification 100×) (B); Squamous Metaplasia (PAS, Hematoxylin-eosin staining, original magnification 200×) (C); Inflammatory cells (PAS, Hematoxylin-eosin staining, original magnification 200×) (D); Atypical cells (PAS, Hematoxylin-eosin staining, original magnification 200×) (E-F).

The presence or absence of goblet cells, inflammatory cells, keratinization; squamous metaplasia, and atypical epithelial cells was evaluated (Figure 1). Goblet cell densities were judged as present when Periodic Acid Schiff (PAS)-positive goblet cells containing mucin were identified in the sample and absent when no goblet cells were visible. Inflammatory cells were judged as present when neutrophils, or eosinophils, monocytes, macrophages, and lymphocytes could be identified. Keratinized cells were identified as enlarged, flattened cells with abundant organophilic (orange/yellow) or eosinophilic (pink) cytoplasm. There may be the presence of keratin filaments and pyknotic nuclei or even enucleated cells. Squamous metaplasia presents as a continuum of chan-ges, including reduction/loss of goblet cells and gradual alterations of nongoblet epithelial cells (i.e., increased keratinization and stratification), as well as cellular enlargement and a decreased nuclear/cytoplasmic ratio^(16)^. Atypical cells were identified by the presence of nuclear enlargement, hyperchromasia, irregular nuclear chromatin, increased NC ratio, and prominent nucleoli^(12^,^17^,^18)^.

## RESULTS

### Clinical profile

Out of the 42 patients examined, IC was performed in 62 eyes of 34 participants (65% females). The mean age of participants was 29.6 ± 17 years (range, 7-62). At baseline examination, 15 of 62 eyes (24%) had a clinical diagnosis of OSSN. The baseline summary findings (Table 1) and detailed observations are tabulated (Table 2-Online supplement).

**Table 1:** Impression cytology: Summary findings of 62 eyes at baseline

Cytology finding^*^	n (%)
Goblet cells	47 (75)
Inflammatory cells	12 (19)
Keratinization	5 (8)
Squamous metaplasia	30 (48)
Atypical epithelial cells	(29)

* Each eye had more than one finding.

**Table 2:** Online supplement. Impression cytology: Detailed findings of 62 eyes at baseline

Case ID	Sex	Age	Eye	OSSN^*^	Goblet cells	Inflammatory cells	Keratinization	Squamous metaplasia	Atypical cells
1	M	43	Right	Yes	Yes	No	Yes	No	Yes
2	F	17	Right	No	Yes	Yes	Yes	Yes	Yes
			Left	No	Yes	Yes	No	No	Yes
3	F	28	Right	No	Yes	No	No	Yes	No
			Left	No	Yes	No	No	Yes	No
4	F	28	Right	No	Yes	No	No	Yes	No
			Left	No	Yes	No	No	Yes	No
5	F	19	Right	No	No	No	No	No	No
			Left	No	Yes	No	No	No	No
6	F	50	Right	No	Yes	No	No	Yes	No
			Left	No	Yes	No	No	Yes	No
7	M	17	Right	No	No	No	No	No	No
8	F	62	Right	No	Yes	Yes	No	No	No
9	M	55	Right	No	Yes	No	No	No	No
			Left	No	Yes	No	No	No	Yes
10	M	20	Right	No	No	Yes	No	Yes	No
			Left	Yes	No	No	No	No	Yes
11	M	29	Right	No	Yes	No	No	Yes	No
			Left	No	Yes	No	No	Yes	No
12	F	11	Right	No	Yes	No	No	Yes	No
			Left	Yes	Yes	No	No	No	Yes
13	F	9	Right	Yes	No	Yes	No	No	Yes
			Left	No	No	Yes	No	Yes	No
14	M	8	Right	No	Yes	No	No	No	No
15	F	26	Right	No	Yes	Yes	No	No	No
			Left	No	Yes	Yes	No	No	No
16	F	23	Right	Yes	Yes	Yes	No	No	Yes
			Left	Yes	No	No	No	No	No
17	M	24	Right	No	Yes	No	No	Yes	No
			Left	No	Yes	No	No	Yes	No
18	F	52	Right	No	Yes	No	No	Yes	No
			Left	Yes	Yes	No	No	Yes	Yes
19	M	32	Right	No	Yes	No	No	No	No
			Left	No	Yes	No	No	No	No
20	M	26	Right	No	Yes	No	Yes	Yes	Yes
			Left	Yes	Yes	No	Yes	Yes	Yes
21	F	27	Right	No	Yes	No	Yes	Yes	Yes
			Left	Yes	No	No	No	Yes	Yes
22	F	9	Right	No	Yes	No	No	No	No
			Left	No	Yes	No	No	No	No
23	F	23	Right	Yes	No	Yes	No	Yes	Yes
			Left	No	No	Yes	No	No	No
24	M	7	Right	No	Yes	No	No	No	No
			Left	No	Yes	No	No	No	No
25	F	17	Right	No	Yes	No	No	No	No
26	F	49	Right	No	Yes	No	No	Yes	No
			Left	No	Yes	No	No	Yes	No
27	M	20	Right	Yes	Yes	No	No	No	No
			Left	No	No	No	No	Yes	No
28	F	45	Right	No	Yes	Yes	No	No	No
			Left	No	Yes	No	No	Yes	No
29	F	61	Right	No	Yes	No	No	No	No
			Left	No	Yes	No	No	No	No
30	F	34	Right	No	Yes	No	No	Yes	No
			Left	No	Yes	No	No	Yes	No
31	F	61	Right	Yes	No	No	No	No	Yes
			Left	Yes	Yes	No	No	Yes	No
32	F	13	Right	Yes	Yes	No	No	No	Yes
			Left	Yes	Yes	No	No	No	Yes
33	M	51	Right	No	No	No	No	Yes	No
			Left	No	No	No	No	Yes	No
34	F	10	Right	No	No	No	No	No	No
			Left	No	No	No	No	No	Yes
Total					47		5	30	18

* Clinical diagnosis of ocular surface squamous neoplasia.

### IC findings

The baseline IC showed goblet cells (47/62, 79%), inflammatory cells (12/62, 19%), keratinization (5/62, 8%), and squamous metaplasia (30/62, 48%). Representative IC findings are shown in figure 1.

### Atypical cells: specificity/ sensitivity

Atypical cells were detected on IC in 18 patients (12 with OSSN and 6 without OSSN). Twelve eyes (19.3%) had atypical cells in IC and had clinical suspicion of OSSN, and three eyes (4.8%) had no atypical cells in IC and had clinical suspicion of OSSN. The detection of atypical cells in IC had 80% sensitivity, 87% specificity, 67% positive predictive value, and 93% negative predictive value for OSSN using clinical diagnosis as the reference standard (Table 3).

**Table 3:** Impression cytology and clinical diagnosis of ocular surface squamous neoplasia: a correlative analysis of 62 eyes

		Clinical diagnosis of ocular surface squamous neoplasia		Total
		Present	Absent	
Atypical cells	Present	12	6	18
	Absent	3	41	44
	Total	15	47	62

Sensitivity = 12/15 (80%).

Specificity = 41/47 (87%).

Positive predictive value = 12/18 (67%).

Negative predictive value = 41/44 (93%).

### Correlation of IC findings with 1-year clinical course

**Figure 2:**
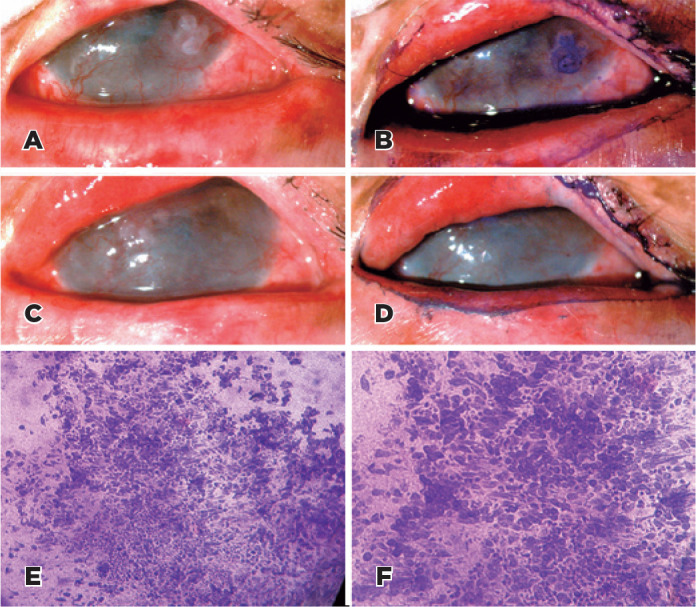
A 23-year-old woman had a history of OSSN in both eyes and had undergone surgery for lower eyelid excision for basal cell carcinoma. Anterior segment slit-lamp photograph demonstrating conjunctival intraepithelial neoplasia (A) and positive staining with toluidine blue 1% (B). The patient was treated with interferon alfa-2b 1 million IU eye drops 4 times daily for 6 months. This led to complete regression as shown by slit-lamp examination (C) and the absence of toluidine blue 1% stain positive area (D). IC was positive for atypical cells (PAS, Hematoxylin-eosin staining, original magnification 100× (E) and 200× (F).

Thirteen participants completed one year of follow-up and repeated the IC examination. After one year, only one patient had residual OSSN despite treatment. The other patients experienced complete remission of OSSN after treatment. No recurrent or new tumors were observed. There were no cases with positive IC (presence of atypical cells) who later developed OSSN. At one-year follow-up, the IC findings were as follows: goblet cells (19/24, 75%), inflammatory cells (2/24, 8%), keratinization (3/24, 12%), and squamous metaplasia (13/24, 54%). The IC was false-positive for atypical cells in one patient (without OSSN) (Table 4). The patient (#23) had undergone successful treatment for OSSN with INF alpha-2b eye drops (1 million IU). At 1-year follow-up assessment, the patient showed no residual lesion on slit-lamp examination and TB staining despite the presence of atypical cells in IC (Figure 2).

**Table 4:** Impression cytology: Longitudinal findings at one-year follow-up. OSSN at 1 year: YES (Residual tumor, Recurrent tumor, New tumor)

Case	Age, gender	Eye	Clinical findings			Impression cytology findings		
			OSSN baseline	OSSN At 1 year	Interval course	Atypical cells Baseline	Atypical cells At 1 year	Status change
2	17, F	Right	No	No	None	Yes	No	Yes
		Left	No	No	None	Yes	No	Yes
14	8, M	Right	No	No	None	No	No	No
15	26, F	Right	No	No	None	No	No	No
		Left	No	No	None	No	No	No
16	23, F	Right	Yes	Yes-Residual tumor	Topical 5 FU	Yes	No	Yes
		Left	Yes	No	Topical 5 FU	No	No	No
18	52, F	Right	No	No	None	No	No	No
		Left	Yes	No	Topical IFN	Yes	No	Yes
22	9, F	Right	No	No	None	No	No	No
		Left	No	No	None	No	No	No
23	23, F	Right	Yes	No	Topical IFN	Yes	Yes	No
		Left	No	No	None	No	No	No
24	7, M	Right	No	No	None	No	No	No
		Left	No	No	None	No	No	No
25	17, M	Right	No	No	None	No	No	No
26	49, F	Right	No	No	None	No	No	No
		Left	No	No	None	No	No	No
29	61, F	Right	No	No	None	No	No	No
		Left	No	No	None	No	No	No
30	34, F	Right	No	No	None	No	No	No
		Left	No	No	None	No	No	No
33	51, M	Right	No	No	None	No	No	No
		Left	No	No	None	No	No	No

OSSN= ocular surface squamous neoplasia.

5 FU= 1% 5-Fluorouracil eye drops.

IFN= Interferon alpha-2b eye drops (1 million IU/mL).

## DISCUSSION

Normal ocular surface epithelium, corneal and conjunctival, is a nonkeratinized multistratified plane consisting of 5-7 layers of polygonal cells. In the conjunctiva, numerous goblet cells also contribute to the tear film’s formation^(19)^. Diseases such as dry eye and ocular cicatricial pemphigoid are characterized by adaptive changes in the epithelium such as pathological transition to nonsecretory keratinized corneal and conjunctival cells, squamous metaplasia, and depletion of goblet cells^(19^-^25)^. In our series of patients with XP, the detection of goblet cells in most eyes (47, 75%) is indicative of the high technical quality of the IC procedure. Approximately 50% (30 eyes) of our patients demonstrated squamous metaplasia, a reflection of the alterations in the surface epithelium secondary to XP.

Histopathology is the gold standard for the diagnosis of OSSN^(26)^. However, only 50% of ophthalmologists perform biopsy to document a pathological diagnosis before starting topical therapy whereas others rely only on the clinical diagnosis possibly due to limited access to pathology services or costs^(5^,^14^,^27)^. IC is a rapid and easy method and when performed by an expert pathologist, it has high accuracy for the initial diagnosis and follow-up of OSSN^(5^,^12^-^14^,^17^,^28)^. IC is a minimally invasive technique that avoids surgical excision of the ocular surface, thereby sparing the corneal stem cells in the limbal area^(12^,^14)^. Therefore, IC offers a safer alternative to repeated surgical biopsies^(12)^. In the present study, pathologic confirmation of the diagnosis was not done to avoid further compromising the surface tissues in this high-risk population that has a tendency for development of new tumors or recurrences^(12)^.

Management of OSSN can be divided into surgical and medical management. A follow-up study conducted in 2012 showed that surgery has remained the mainstay of therapy, but that there had been a significant increase in the use of topical therapy. With the advent of less invasive diagnostic modalities such as AS-OCT, confocal microscopy, and IC, there has been a shift toward less invasive management options. The three most commonly used topical treatments are interferon-α2b (IFN), 5-fluo-rouracil (5FU), and mitomycin-C (MMC). All three have shown similar outcomes in terms of tumor resolution rates and recurrence. The main differences relate to cost, storage, and side effect profile. Topical therapy avoids the risks associated with surgery and offers the benefit of treating the entire ocular surface. For larger tumors that extend onto the cornea, topical therapy avoids the risk of intraoperative corneal damage which may have significant visual consequences. Smaller tumors can be managed surgically or medically with equal success. Large tumors are preferentially managed by medical therapy to reduce surgical morbidity^(29).^

Although IC showed a moderate positive predictive value (67%) for diagnosis of OSSN in patients with XP, it had a high sensitivity (80%), specificity (87%), and negative predictive value (93%). In other words, IC that is negative for atypical cells can be informative in excluding OSSN.

It should be emphasized that the technique can be repeated, and patients can be followed until atypical cells are detected. It is important to note that IC is not a substitute for a possible biopsy for arriving at a definitive diagnosis and to guide appropriate treatment. In high-risk individuals, such as patients with XP, IC can be integrated into long-term management, as it may avoid unnecessary surgical biopsies.
